# Oncogenic K-ras confers SAHA resistance by up-regulating HDAC6 and c-myc expression

**DOI:** 10.18632/oncotarget.7134

**Published:** 2016-02-02

**Authors:** Qun Wang, Rong Tan, Xin Zhu, Yi Zhang, Zhiping Tan, Bing Su, Yu Li

**Affiliations:** ^1^ Shanghai Institute of Immunology, Shanghai Jiaotong University School of Medicine, Shanghai, China; ^2^ Xiangya Hospital, Central South University, Hunan, China; ^3^ Clinical Center for Gene Diagnosis and Therapy of The State Key Laboratory of Medical Genetics, Hunan, China; ^4^ Department of Immunobiology and The Vascular Biology and Therapeutics Program, Yale School of Medicine, New Haven, Connecticut, USA

**Keywords:** K-ras, HDAC inhibitor, SAHA resistance

## Abstract

Histone deacetylase inhibitors (HDIs) represent a new class of anticancer drugs. Suberoylanilide hydroxamic acid (SAHA), the first HDI approved for the treatment of cutaneous T cell lymphoma (CTCL), is currently being tested in clinical trials for other cancers. However, SAHA has been ineffective against solid tumors in many clinical trials. A better understanding of molecular mechanisms of SAHA resistance may provide the basis for improved patient selection and the enhancement of clinical efficacy. Here we demonstrate that oncogenic K-ras contributes to SAHA resistance by upregulating HDAC6 and c-myc expression. We find that the high levels of HDAC6 expression are associated with activated K-ras mutant in colon cancer patients. And expressions of HDAC6 and c-myc are increased in fibroblasts transformed with activated K-ras. Surprisingly, we find that activated K-ras transformed cells are more resistant to SAHA inhibition on cell growth and anchorage-independent colony formation. We show that a K-ras inhibitor sensitizes K-ras mutated lung cancer cells to SAHA induced growth inhibition. We also find that mutant K-ras induces HDAC6 expression by a MAP kinase dependent pathway. Our study suggests that combined treatment with SAHA and K-ras inhibitors may represent an effective strategy to overcome SAHA resistance.

## INTRODUCTION

Histone deacetylases (HDACs) are a family of enzymes that remove acetyl groups from lysine residues. Besides the well-established role of HDACs in regulating histone acetylation and gene transcription, recent studies have revealed that HDACs have diverse functions in a much broader array of biological processes [1]. The best example is HDAC6, which has been implicated in regulation of microtubules, growth factor-induced chemotaxis, misfolded protein stress response and tumor invasion [2–7]. Our previous study showed that HDAC6 is involved in EGF-induced β-catenin nuclear localization and activation of c-myc [8]. HDAC6 deacetylates β-catenin at lysine 49 and promotes its nuclear import. Inactivation of HDAC6 decreases c-myc expression, leading to inhibition of tumor cell proliferation. Consistent with our study, Lee *et al.* found that HDAC6 is required for efficient oncogene-induced tumorigenesis in mouse, and that fibroblasts deficient in HDAC6 are more resistant to both oncogenic Ras and ErbB2-induced transformation [9]. On the other hand, we also found that overexpression of HDAC6 leads to increased level of c-myc, suggesting HDAC6 could play a positive role in tumorigenesis [8].

HDAC inhibitors (HDIs) represent a promising new class of anticancer drugs. SAHA (Vorinostat) has been approved for the treatment of cutaneous T cell lymphoma (CTCL), and it is currently being evaluated in other cancer types [10].

Recent studies also show that the combination of HDIs and alkylating agents exhibit efficient anti-proliferative activity on myeloid leukemia cells [11]. HDIs have been shown to induce differentiation, cell cycle arrest, autophagy and apoptosis in a variety of tumor cell lines, inhibit tumor growth in animal models, and show antitumor activity in clinical trials [12, 13]. However, SAHA has been ineffective against solid tumors in many clinical trials, including colorectal and non-small cell lung cancers. Poor response to treatment could be linked to systemic factors like pharmacokinetics or to tumor-specific factors both at the level of the malignant cell or the tumor microenvironment [14]. It has been shown that overexpression of HDAC1 in melanoma cells was sufficient to confer HDI resistance [15]. An inactivating mutation in HDAC2 was identified in various human colon and endometrial cancer cell lines. Treatment of HDAC2-deficient cells with TSA failed to induce histone acetylation and inhibit proliferation [16]. HDIs induce apoptosis in a variety of malignant cells. And it has been shown that overexpression of antiapoptotic Bcl-2 is sufficient to confer HDIs resistance [17]. Because Bcl-2 overexpression occurs in leukemias and lymphomas, it is possible that it may play a role in clinical response to HDIs. Furthermore, the antiapoptotic transcription nuclear factor κB (NF-κB) has also been identified as a mediator of resistance to HDI treatment. It has been shown that the activation of NF-κB by HDIs interferes with their ability to trigger cell death in non–small cell lung cancer and leukemia cell lines. And inhibition of NF-κB activation sensitizes the malignant cells to death in response to inhibition of HDACs [18].

Activating mutations of K-ras are found in approximately 30% of human cancers. K-ras is commonly mutated at codon 12 or 13 [19, 20]. GTP-bound K-ras converts extracellular stimuli into intracellular signaling cascades underlying diverse cellular activities such as cell proliferation and survival. The Ras-bound GTP is then hydrolyzed to GDP, resulting in termination of signaling. Thus, K-Ras acts as a molecular switch to regulate the RAF-MEK-ERK and the PI3K-Akt pathways, and mutations in K-Ras favoring its active, GTP-bound forms will lead to aberrant intracellular signaling, resulting in uncontrolled cell proliferation and survival in tumors. Besides its well studied role in tumorigenesis, Ras activating mutations are also involved in antitumor drug resistance in lung and colon cancers. It has been shown that clinical responses to cetuximab, an anti-EGFR antibody approved for colon cancer treatment, are restricted to patients with wild-type K-ras tumors [21]. K-ras mutations are used to predict the lack of clinical benefit from cetuximab treatment in colon cancer and to select colon cancer patients for the antibody therapy.

Here we show that oncogenic K-ras contributes to SAHA resistance. We find that activated K-ras mutants are associated with the high level of HDAC6 in colon cancer patients. Our previous study showed that overexpression of HDAC6 could promote tumor cell growth by activating oncogene c-myc. Consistently, we find that expressions of both HDAC6 and c-myc are significantly increased in fibroblasts transformed with an activated K-ras mutant. Importantly, we find that K-ras transformed cells are more resistant to SAHA inhibition on cell growth and anchorage-independent colony formation. We show that a K-ras mutant-specific inhibitor sensitizes cancer cells to SAHA induced growth inhibition. We also show that K-ras can induce HDAC6 expression by a MAP kinase dependent pathway, and that SAHA suppresses c-myc expression and tumor growth in K-ras transformed cells. Our study demonstrates that K-ras confers SAHA resistance by upregulating HDAC6 and c-myc expression and suggests that a combined treatment with SAHA and K-ras inhibitors may represent a useful strategy to overcome SAHA resistance induced by oncogenic K-ras.

## RESULTS

### Association of K-ras mutation and high expression of HDAC6 in colon cancer patients

**Figure 1:**
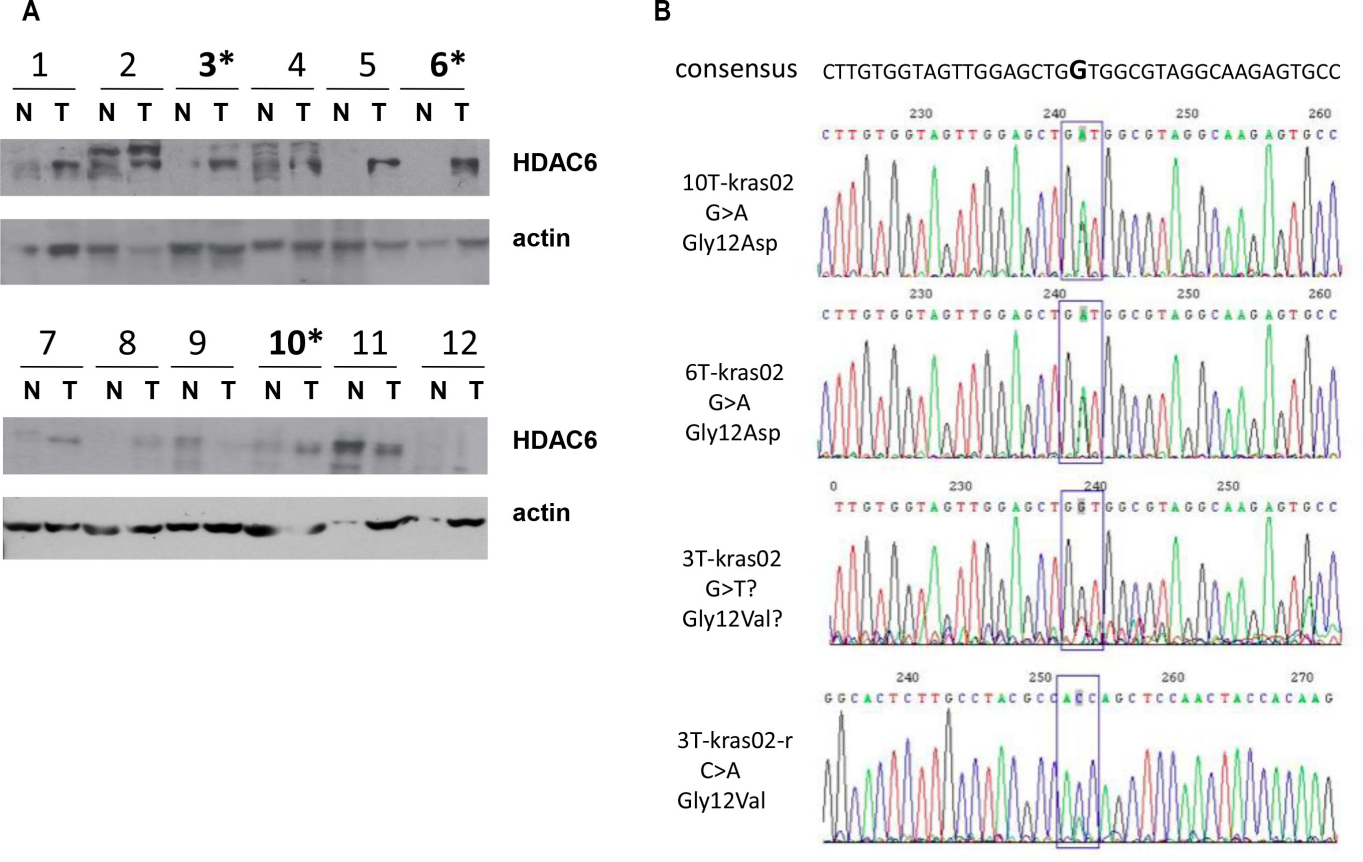
Association of K-ras mutation and high expression of HDAC6 in colon cancer patients (**A**) Immunoblotting of colon tumor tissues. Samples from tumor (T) and adjacent normal (N) tissues of 12 patients were immunoblotted with antibodies indicated. (**B**) Sequencing of exon1 of K-ras from DNA extracted from tumor tissues obtained from patients 3, 6 and 10 in Figure 1A, G > A and G > T mutations are indicated.

Our previous study showed that overexpression of HDAC6 leads to increased level of c-myc expression, suggesting HDAC6 could play a positive role in tumorigenesis. To test this hypothesis, both human colon cancer tissues and adjacent normal tissues (*n* = 12) were collected. Western blotting showed higher expression of HDAC6 protein in 7 (59%) tumor tissues compared to controls (Figure 1A), suggesting that HDAC6 may be involved in human colon cancer development. Since K-ras is commonly mutated in colorectal carcinoma, we also analyzed the status of K-ras from the same patient samples by DNA sequencing. We identified G12D K-ras mutation in tumor tissues from patient No. 6 and 10. In patient No. 3, we identified both wild type and G12V K-ras, suggesting that the mutation is heterozygous or it is difficult to microdissect pure tumor. To confirm the K-ras status in patient No.3, we sequenced both strands of K-ras DNA and confirmed G12V mutation (Figure 1B). Together, we identified K-ras mutations in 3 of 12 (25%) tumor tissues. Interestingly, all 3 tumor tissues with activated K-ras mutants showed increased level of HDAC6, suggesting a positive role of K-ras on HDAC6 expression.

### HDAC6 and c-myc expression are induced by K-ras mutation in fibroblasts

**Figure 2:**
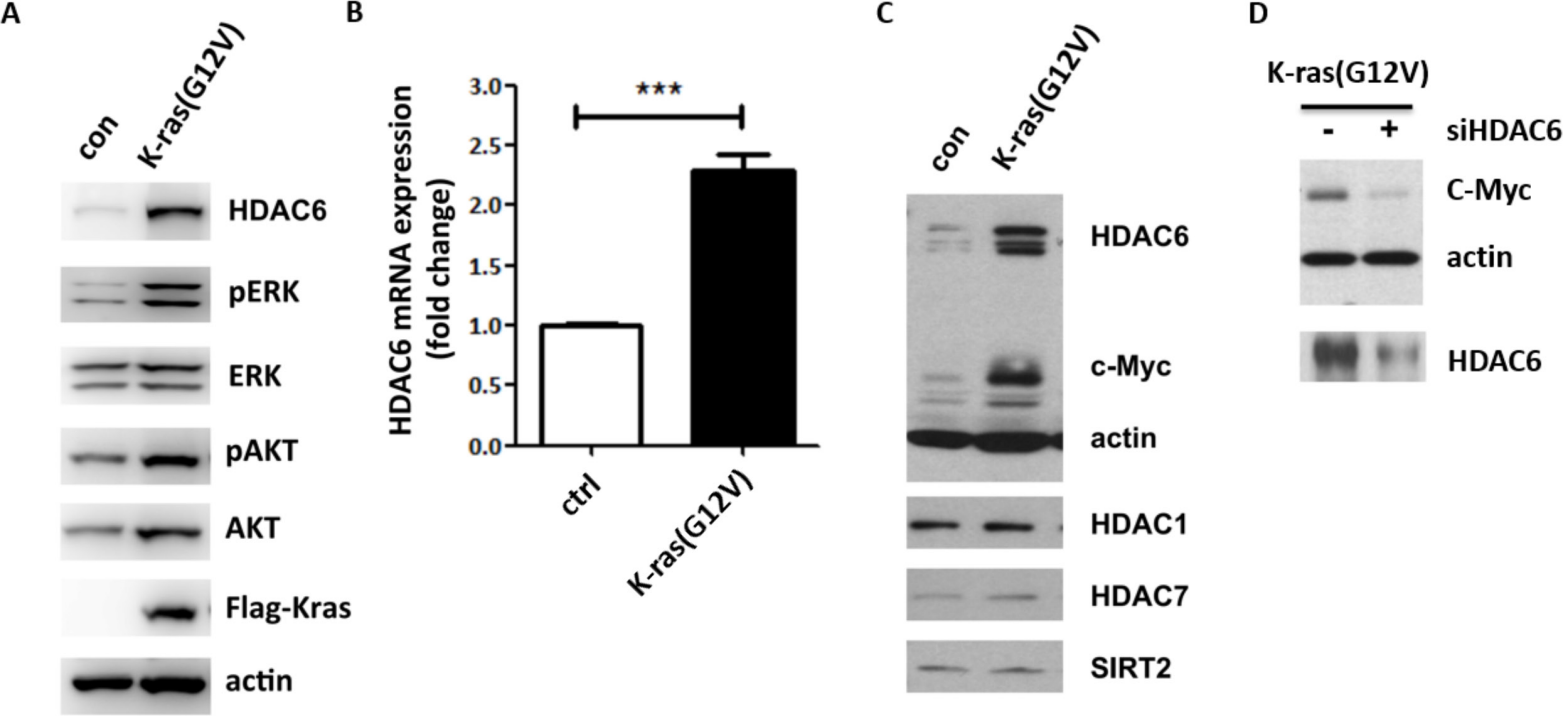
HDAC6 and c-myc expressions are induced by activated K-ras in fibroblasts (**A**) Immunoblotting of 10T1/2 MEF cells stably transfected with K-ras G12V. Cell lysates were collected and immunoblotted with antibodies indicated. (**B**) Analysis of HDAC6 expression by RT-PCR. RNA was extracted from 10T1/2 and 10T1/2 Kras cells. HDAC6 mRNA expression was analyzed following the manufacturer's instruction. (**C**) Immunoblotting of 10T1/2 K-ras cells. Cell lysates were collected and immunoblotted with antibodies indicated. (**D**) Immunoblotting of 10T1/2 K-ras cells transfected with HDAC6 siRNA. 48 h after transfection, lysates were immunoblotted with the antibodies indicated.

The positive correlation between K-ras mutants and high level of HDAC6 expression in colon cancer patients prompted us to test if K-ras mutants induce HDAC6 expression. To this end, we introduced an activated K-ras mutant into 10T1/2 fibroblast cells and examined HDAC6 protein level. Immunoblotting showed that HDAC6 was increased in 10T1/2 cells stably transfected with K-ras G12V mutation (Figure 2A). As expected, phosphorylation of ERK and AKT were also increased in K-ras transfected cells. To test if K-ras regulated HDAC6 expression at the transcription level, we analyzed HDAC6 mRNA level by quantitative real-time PCR (RT-PCR). As shown in Figure 2B, HDAC6 mRNA level was increased by > 2 fold in 10T1/2 cells stably expressing K-ras, further supporting a potential role of HDAC6 in K-ras induced tumorigenesis. Then we asked if K-ras had the same effect on expression of other members of HDAC family. As shown in Figure 2C, we found that the expression levels of other HDACs, such as HDAC1, HDAC7 and SIRT2, were not affected by K-ras mutant, suggesting that HDAC6 may be a specific effector of K-ras.

Since we found that overexpression of HDAC6 promotes β-catenin nuclear translocation and transcription of its target gene like c-myc, we asked if K-ras induced HDAC6 expression also enhanced c-myc expression. As expected, c-myc level was indeed increased in 10T1/2 cells expressing the K-ras mutant (Figure 2C). To further confirm that K-ras induced c-myc expression is dependent on HDAC6, HDAC6 siRNA was introduced into 10T1/2 K-ras cells and as shown in Figure 2D, HDAC6 siRNA inhibited c-myc expression in 10T1/2 K-ras cells, suggesting a positive role for HDAC6 in K-ras induced c-myc expression.

### Oncogenic K-ras transformed cells are more resistant to SAHA induced growth inhibition

**Figure 3:**
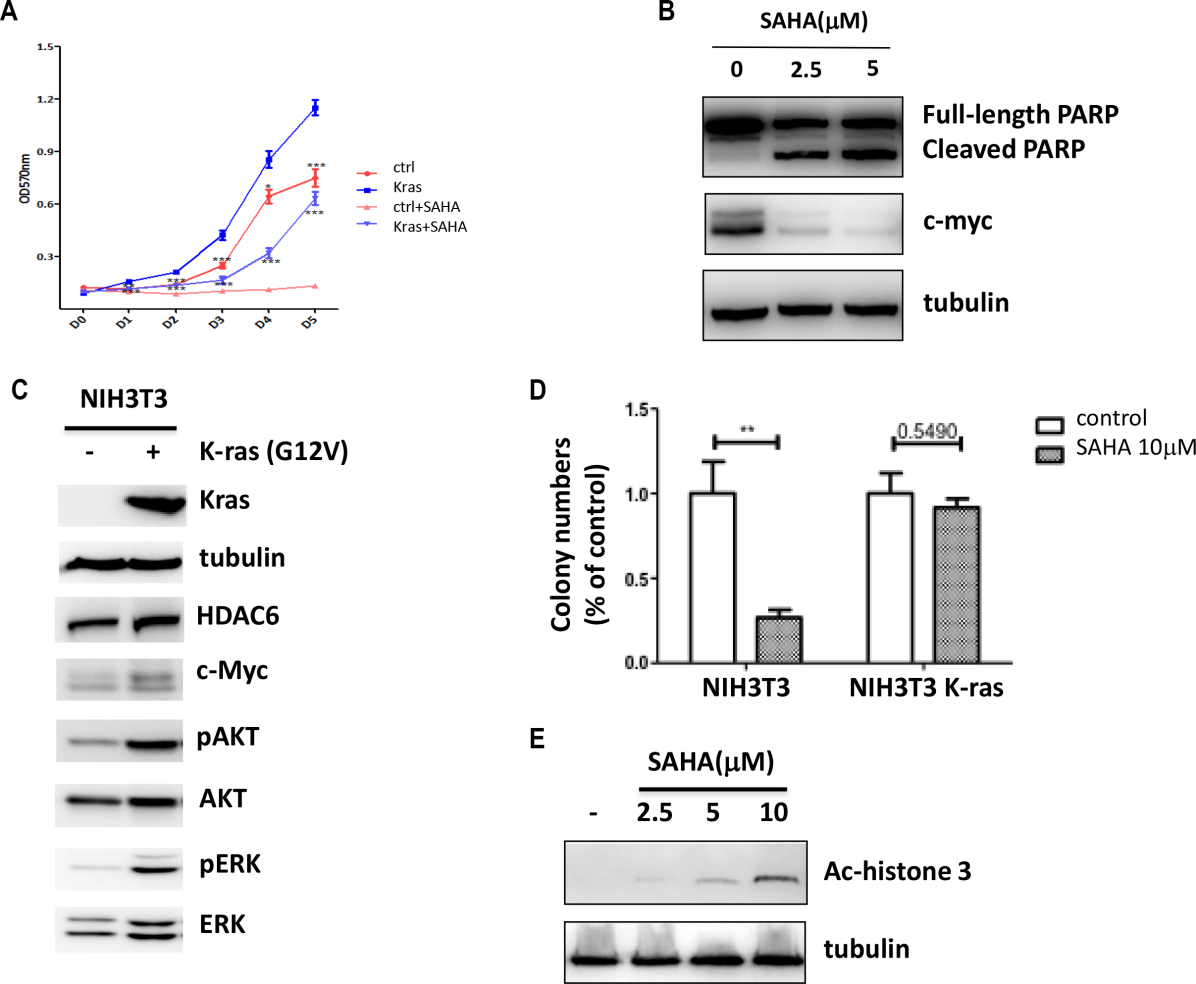
Oncogenic K-ras confers SAHA resistance (**A**) Proliferation of 10T1/2 K-ras G12V cells after SAHA (2.5 mM) treatment. Cells were collected and analyzed by MTT assay. Results are from three independent experiments. (**B**) Immunoblotting of HCT116 cells treated with SAHA for 24 hrs. Cell lysates were collected and immunoblotted with antibodies indicated. (**C**) Immunoblotting of NIH3T3 cells stably transfected with K-ras G12V. Cell lysates were collected and immunoblotted with antibodies indicated. (**D**) Anchorage-independent colony formation of NIH3T3 cells. Both wide-type and K-ras transformed NIH3T3 Cells were treated SAHA (10 mM) and colony formation was determined by soft agar assay. Results are from three independent experiments. (**E**) NIH3T3 cells were treated with SAHA of different concentrations and immunoblotted with ac-histone3. 10 mM of SAHA was chosen for the soft agar assay.

The observation that K-ras induces HDAC6 dependent c-myc expression prompted us to test if HDAC inhibitors could prevent K-ras induced tumorigenesis. To this end, we examined the effect of SAHA on K-ras induced tumor cell growth. As shown in Figure 3A introduction of the K-ras mutant into 10T1/2 cells promoted cell growth and this K-ras induced cell growth was inhibited by SAHA treatment. Surprisingly, when we compared both wild-type and K-ras transformed 10T1/2 cells treated with SAHA, we found that the effect of SAHA on growth inhibition was partially reversed by K-ras mutant. To test if apoptosis is induced by SAHA treatment in cancer cells with K-ras mutant, we examined the SAHA effect on HCT116, a colon cancer cell line which has the G13D activating mutation in one of the K-ras alleles. As shown in Figure 3B, SAHA treatment increased PARP cleavage, suggesting that SAHA could induce apoptosis in cancer cells with K-ras mutant.

Next, we assessed anchorage-independent colony formation to analyze the effect of SAHA in cells with the K-ras mutant. Since we did not observe any colony formation in wild-type 10T1/2 cells (data not shown), we used NIH3T3 cells instead in this assay. To this end, wild-type NIH3T3 cells were transduced with K-ras G12V, and the effects of SAHA were assessed with respect to colony formation. Consistently, we found that HDAC6 and c-myc protein levels were increased in NIH3T3 cells stably transfected with K-ras mutant, although the basal level of HDAC6 expression was increased in wild-type NIH3T3 cells relative to 10T1/2 MEF cells (Figure 3C). SAHA treatment of wild-type NIH3T3 cells resulted in the inhibition on colony formation (Figure 3D and 3E). In contrast, when we treated NIH3T3 K-ras cells with SAHA, more colonies were formed than SAHA treated wild-type NIH3T3 cells. Together, these results show that cells with K-ras mutant are more resistant to SAHA induced growth inhibition.

### Inhibition of K-ras sensitizes cancer cells to SAHA treatment

**Figure 4:**
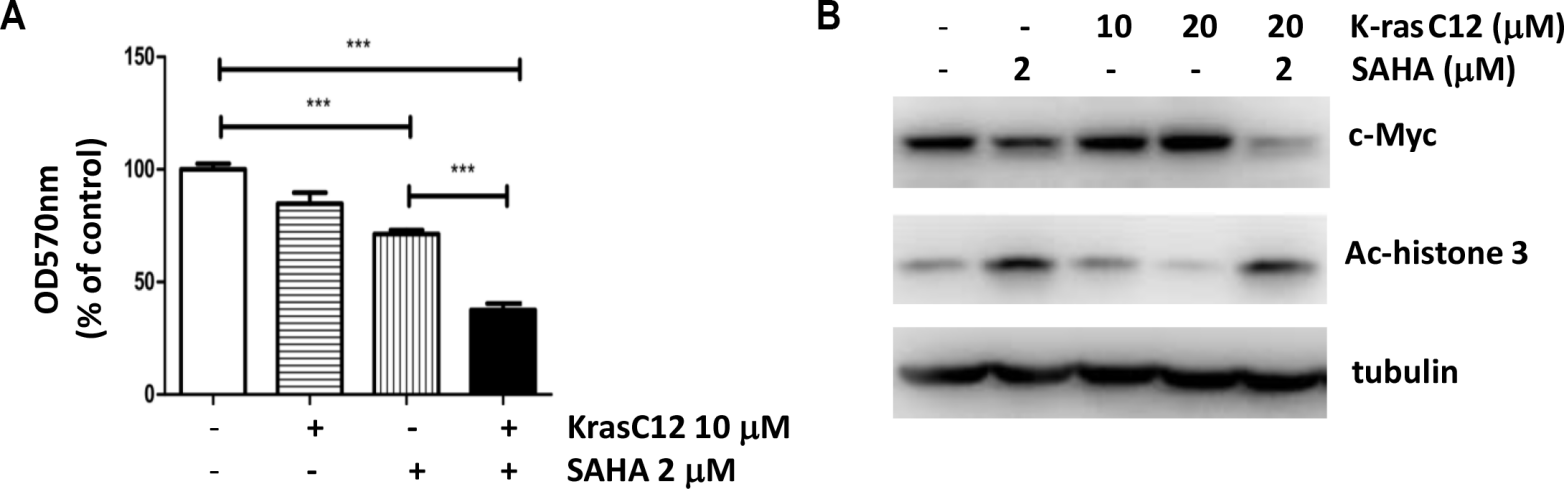
Inhibition of K-ras sensitizes cancer cells to SAHA treatment (**A**) Proliferation of Calu-1 cells after SAHA and K-ras inhibitor (K-ras C12) treatment. Calu-1 cells, containing an activating G12C K-ras mutation, were treated with SAHA (2.5 mM) and K-ras C12 (10 mM) for 24 h and analyzed as in Figure 1A. (**B**) Immunoblotting of Calu-1 cells treated with inhibitors. Cells were treated with inhibitors as indicated for 24 hrs. Cell lysates were collected and immunoblotted with antibodies indicated.

We then asked whether the inhibition of K-ras activity enhances the growth inhibitory effects of SAHA. To test this, we examined the effect of SAHA and a K-ras inhibitor on growth inhibition of Calu-1 cells. Calu-1 is a lung cancer cell line that contains a K-ras G12C activating mutation. And an irreversible, mutant-specific K-ras inhibitor (K-ras inhibitor compound 12, K-ras C12) was developed to target the G12C mutant [22]. As shown in Figure 4A, both SAHA and K-ras C12 treatment resulted in ~30% and ~20% decrease in cell number, whereas the combination of SAHA and K-ras C12 for 24 h reduced cell number by ~70%, suggesting that treatment of K-ras C12 sensitizes cells to SAHA induced growth inhibition. Consistent with previous work, c-myc expression was inhibited by SAHA in Calu-1 cells (Figure 4B). More importantly, although K-ras inhibitor K-ras C12 alone had no effect on the protein level of c-myc, treatment of Calu-1 cells with both SAHA and K-ras C12 gave additive effect on inhibition of c-myc expression (Figure 4B). These results indicate that inhibition of K-ras activity can further enhance the sensitivity of Calu-1 to SAHA.

### K-ras induces HDAC6 and c-myc expression through a MAPK dependent pathway

**Figure 5:**
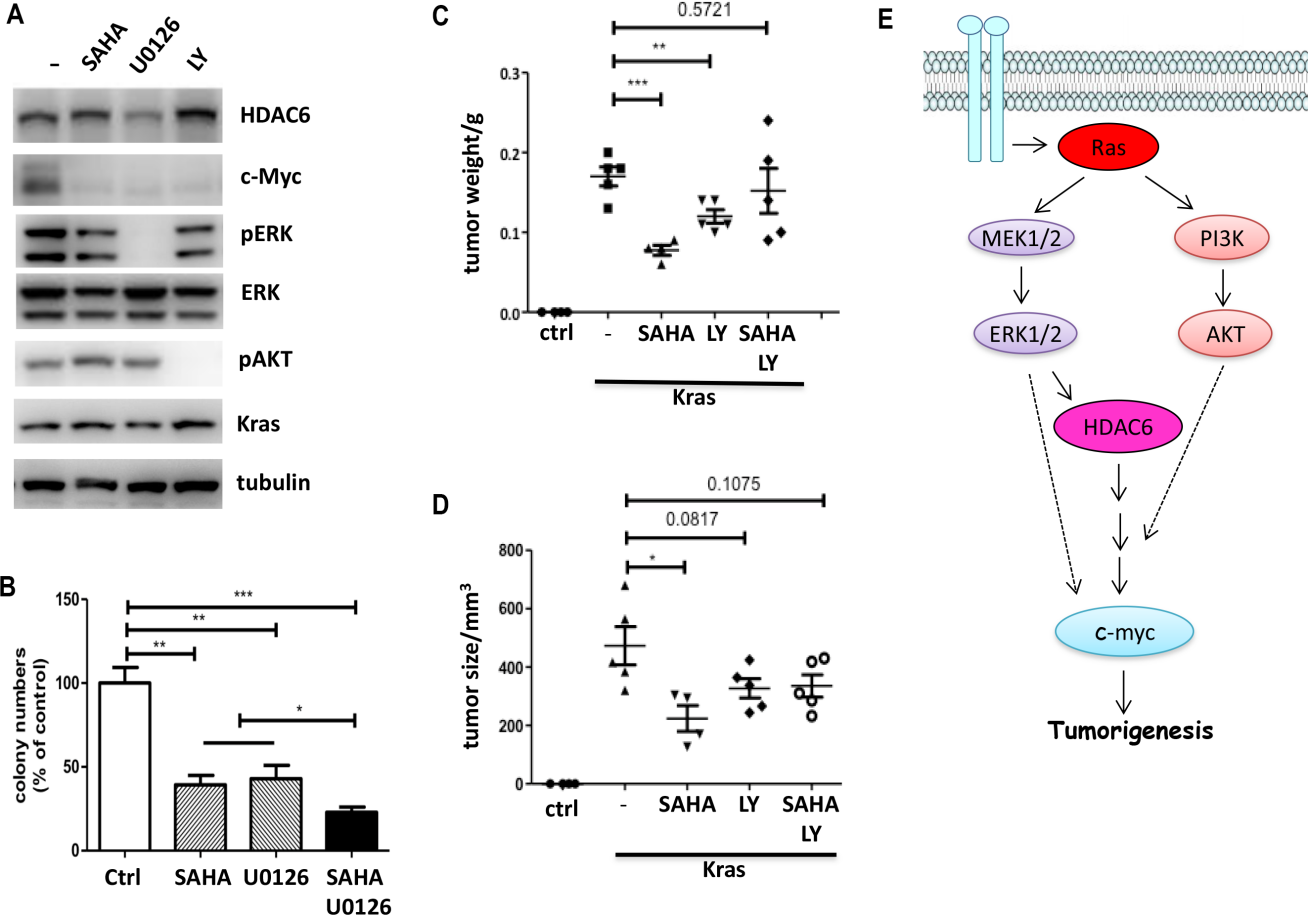
K-ras induced HDAC6 expression is mediated by MAPK signaling (**A**) Immunoblotting of 10T1/2 K-ras cells treated with inhibitors. Cells were treated with inhibitors as indicated. Cell lysates were collected and immunoblotted with antibodies indicated. (**B**) Anchorage-independent colony formation of HCT116 cells. HCT116 Cells were treated with SAHA (1 mM) and U0126 (10 mM). Colony formation was determined by soft agar assay. Results are from three independent experiments. (**C**) and (**D**) Effect of SAHA and PI3K inhibitor on Xenograft tumor growth. 10T1/2 K-ras cells (1 × 10^6^) were injected subcutaneously into nude mice (*n* = 5 per group). 3 days after injection, SAHA (50 mg/kg) and LY294002 (25 mg/kg) were administrated. Mice were euthanized at day 15, and tumors were measured (5C) and weighed (5D). (**E**) A working model of K-ras induced HDAC6 and c-myc activation.

How does K-ras regulate HDAC6 and c-myc expression? It is well known that K-ras acts as a molecular switch to regulate the RAF-MEK-ERK and the PI3K-Akt signaling pathways. Therefore, we asked whether the MAPK and PI3K/Akt pathways regulate HDAC6 and c-myc expression. 10T1/2 K-ras cells were treated with inhibitors of PI3K (LY294002), MAPK (U0126) and SAHA. As shown in Figure 5A, HDAC6 expression was inhibited by U0126, suggesting that the MAPK pathway is involved in K-ras induced HDAC6 expression. We further compared the effect of SAHA and U0126 on colony formation in HCT116, which has a k-ras activating mutation. As shown in Figure 5B, both SAHA and U0126 exhibited similar effects on colony formation inhibition, and the combination of SAHA and U0126 gave the additive effect, suggesting that MAPK could also activate a HDAC6 independent pathway to promote cell growth in K-ras mutant cancer cells. Curiously, although the PI3K inhibitor had no effect on HDAC6 expression, it blocked c-myc expression (Figure 5A), suggesting that K-ras induced c-myc expression could be regulated by HDAC6 and PI3K pathways independently, or that both HDAC6 and PI3K contribute to c-myc induction and therefore tumor cell growth. To distinguish between these two possibilities, we examined the effect of SAHA and PI3K inhibition on tumor cell growth using a xenograft model. Both 10T1/2 and 10T1/2 K-ras cells were inoculated s.c into nude mice and tumor growth was measured over time. As shown in Figure 5C and 5D, control 10T1/2 cells did not form palpable tumors (0/5), whereas 10T1/2 K-ras cells developed rapidly growing tumors in nude mice. Most of tumors derived from 10T1/2 k-ras cells reached a size of 0.5 cm^3^ within 2 weeks of injection. Both SAHA and LY294002 treatment reduced the tumor sizes. The combination of SAHA and LY294002 yielded no enhanced inhibition of tumor growth, suggesting that HDAC6 and PI3K contribute to the same pathway, which leads to c-myc expression and tumor growth.

Based on these finding, we propose a model whereby oncogenic K-ras could confer SAHA resistance by upregulating HDAC6 expression through the MAPK signaling, resulting in activation of the downstream oncogene c-myc (Figure 5E). Furthermore, the PI3K pathway also contributes to K-ras induced c-myc expression and tumor growth. Therefore, a positive association between K-ras mutation and elevated HDAC6 and c-myc expression is presumably involved in SAHA resistance.

## DISCUSSION

In this study, we find that HDAC6 protein level is significant increased in 7 of 12 (59%) tumor tissues compared to controls in colon cancer patients. We also find that the activated K-ras mutants are associated with high levels of HDAC6 expression in these patients. We demonstrate that both HDAC6 and c-myc are induced by an activated K-ras mutant and inhibition of HDAC6, either by HDAC6 siRNA or SAHA, blocks K-ras induced c-myc expression and tumor growth. However, it is unclear what causes HDAC6 up-regulation in other colon cancer patients without K-ras activating mutations. Elevated levels of HDAC6 have also been reported in acute myeloid leukemia and some breast cancers [23–25]. In breast cancer cell lines, HDAC6 was shown to be regulated by estrogen [24]. Interestingly, breast cancer patients with high level of HDAC6 had a favorable response to anti-estrogen tamoxifen treatment, suggesting a role of HDAC6 in estrogen-induced tumorigenesis [25].

Most importantly, our study showed that K-ras transformed cells are more resistant to SAHA treatment. Because of its promising results in the treatment of CTCL, SAHA has been tested in many clinical trials to assess its efficacy against different solid tumors, including colorectal and non-small cell lung cancers. Disappointingly, SAHA has not been effective in clinical trials involving those solid tumors. Since K-ras mutations are especially prevalent in colorectal carcinoma (40–45% of cases) and non-small cell lung cancer (NSCLC) (16–40%), our study suggests that testing for K-ras mutations could be very important for patient selection in the future clinical trials of SAHA treatment. In support of this idea, we find that inhibition of K-ras activity enhances cell growth inhibition by SAHA, suggesting a combinational treatment with SAHA and K-ras inhibitor may confer additional clinical benefit.

## MATERIALS AND METHODS

### Cell culture

C3H10T 1/2 (10T1/2 MEF) cell was from Cellbank in Shanghai College of life science, Chinese academy of sciences. NIH3T3 and HCT116 were purchased from American Type Culture Collection (ATCC, USA). The cells were cultured in Dulbecco's modified Eagle's medium supplemented with 10% fetal bovine serum. Lung cancer cell line Calu-1 was a generous gift from Dr. Jiong Deng and was maintained in McCoy's 5A (Modified) medium (Life technologies) wish 10% FBS.

The K-ras mutation in Calu-1 cells was confirmed by sequencing.

### Reagents

Anti-HDAC6, c-myc, AKT, pAKT, ERK1/2 and pERK antibodies were purchased from Cell Signaling Technology (CST). Anti-FLAG was from SIGMA.

Anti-actin was purchased from Zen BioScience (Chengdu). Anti-tubulin antibody was made in our lab. Vorinostat (SAHA) was obtained as a gift from Dr. MingYao Liu in East China Normal University and was also purchased from Selleckchem. since TSA is not mentioned in the text. K-ras(G12C) inhibitor 12 was purchased from Selleckchem. PI3K inhibitor LY294002 was from Abcam.

### Expression K-ras G12V in 10T1/2 MEF and NIH3T3 cells

The plasmid pBabe-Kras G12V was a gift from Dr. Hongbin Ji at Institute of Biochemistry and Cell biology, SIBS, CAS. The K-ras mutant was subcoloned to vector pLVX-flag-SBP to constitute the plasmid pLVX-flag-Kras G12V and was transfected to 293T cells to obtain lentivirus which were then used to infect 10T1/2 MEF and NIH3T3 cells. The transfected cells were selected with 1.2 μg/ml puromycin (SIGMA) to generate a cell pool stably expressing Kras G12V.

### DNA sequencing of K-ras

The entire coding regions, including the flanking intronic sequences of *KRAS* were amplified with polymerase chain reaction (PCR; primer sequences upon request). Sequences of the PCR products were determined using the ABI 3100 Genetic Analyzer (ABI, Foster City, CA).

### RNA extraction and real time PCR analysis

All RNA was purified with Trizol (Thermo) and cDNA was reverse transcripted with TaKaRa Kit according to the manufacturer's instruction. And Q-PCR was performed by using SYBR^®^ Green PCR Master Mix (Applied Biosystems) and ABI ViiA™ 7 system. The relative mRNA expression was presented using the comparative Ct method (2^(−ΔΔCt)^) and two-tailed Student's *t* test was used for analysing the difference. ***P < 0.001 by Student's *t* test.

### Cell proliferation assay

The cell lines were cultured in 96-well plates at the density of 1000 cell/well with 100 μl/well medium with either DMSO or inhibitors. 12 h after the cells had adhered, 20 μl of 5 mg/ml Thiazolyl Blue Tetrazolium Blue was added (MTT, SIGMA). After incubation at 37°C for 3 h, the liquid was removed. 170 μl/well of DMSO was added to dissolve the MTT formazan blue crystals and kept at 37°C for 15 min. And the intensity was measured colorimetrically at 570 nm.

### Soft agar colony formation assay

1,000 stable transfected 10T1/2 MEF, NIH3T3 or HCT116 cells were resuspended in 0.35% agarose gel with 10% FBS DMEM and plated on 0.7% agarose containing DMEM (500 μl/well) in 24-well plate respectively, 4 repeats for each group. Cells were incubated at 37°C, 200 μl of medium with indicated drugs were added every other day. After 10~15 days, the colony number were counted and take photographs. The difference of numbers was statistically analyzed using Student's *t* test.

### Tumor xenografts in nude mice

4-week-old female BALB/cASlac-nu mice were purchased from SRLC Laboratory Animal, and assigned to 5 groups of ctrl, Kras, Kras + SAHA, Kras + LY294002 and Kras + SAHA + LY294002. All animal studies were carried out under the regulation of Shanghai Jiaotong University Medical School. Exponentially growing cells were collected, washed twice and resuspended in PBS, and 1 × 10^6^ cells were injected subcutaneously into mice (*n* = 5 per group). 3 days after injection, the mice were i.p administrated with DMSO, SAHA (50 mg/kg), LY294002 (25 mg/kg) and SAHA + LY294002 respectively in every 3 days. Tumor weight and size were measured. Mice were sacrificed at day 15 and the tumors were dissected for the subsequent analysis.
